# Preparation of fatty acid solutions for investigating lipid signaling, metabolism, and lipid droplets

**DOI:** 10.1093/procel/pwae068

**Published:** 2024-12-17

**Authors:** Shuyan Zhang, Mengwei Zhang, Shimeng Xu, Xiaochuan Fu, Qiumin Liao, Bin Pan, Liujuan Cui, Pingsheng Liu

**Affiliations:** National Key Laboratory of Intelligent Tracking and Forecasting for Infectious Diseases, Beijing Ditan Hospital, Capital Medical University, Beijing 100015, China; Beijing Key Laboratory of Emerging Infectious Diseases, Institute of Infectious Diseases, Beijing Ditan Hospital, Capital Medical University, Beijing 100015, China; Beijing Institute of Infectious Diseases, Beijing 100015, China; National Center for Infectious Diseases, Beijing Ditan Hospital, Capital Medical University, Beijing 100015, China; Institute of Biophysics, Chinese Academy of Sciences, Beijing 100101, China; Institute of Biophysics, Chinese Academy of Sciences, Beijing 100101, China; College of Life Science and Technology, Huazhong University of Science and Technology, Wuhan 430074, China; University of Chinese Academy of Sciences, Beijing 100049, China; Institute of Biophysics, Chinese Academy of Sciences, Beijing 100101, China; Institute of Biophysics, Chinese Academy of Sciences, Beijing 100101, China; National Key Laboratory of Intelligent Tracking and Forecasting for Infectious Diseases, Beijing Ditan Hospital, Capital Medical University, Beijing 100015, China; Beijing Key Laboratory of Emerging Infectious Diseases, Institute of Infectious Diseases, Beijing Ditan Hospital, Capital Medical University, Beijing 100015, China; Beijing Institute of Infectious Diseases, Beijing 100015, China; National Center for Infectious Diseases, Beijing Ditan Hospital, Capital Medical University, Beijing 100015, China; Institute of Biophysics, Chinese Academy of Sciences, Beijing 100101, China; Institute of Biophysics, Chinese Academy of Sciences, Beijing 100101, China; Institute of Biophysics, Chinese Academy of Sciences, Beijing 100101, China; University of Chinese Academy of Sciences, Beijing 100049, China

**Dear Editor,**

Fatty acids (FAs) are amphipathic molecules composed of a hydrocarbon chain with a terminal carboxyl group. While short-chain FAs have some solubility in water due to their hydrophilic carboxylic acid group, most FAs are insoluble in water because of their long, nonpolar hydrocarbon chains (Nelson et al., 2008). Furthermore, the solubility of FAs in water decreases as the hydrocarbon chain lengthens and the number of double bonds decreases (Nelson et al., 2008).

FAs play crucial roles in membrane structure, energy storage and production, carbon source, protein modification, and cell signaling. Unesterified FAs in blood plasma and extracellular fluids, important factors linked to various metabolic disorders in humans, predominantly bind non-covalently to the carrier protein albumin (Khan et al., 2023; Nelson et al., 2008; van der Vusse, 2009). In addition, FAs esterified in triacylglycerols (TAGs) are mainly present in chylomicrons (CM) and very low-density lipoproteins, where they can be released by lipoprotein lipase (LPL). FAs act as metabolic regulators by signaling through membrane FA receptors. They are also taken up into cells via passive diffusion or protein-mediated translocation, initiating specific metabolic processes (Samovski et al., 2023). FAs also serve as integral components of phospholipids in cellular membranes (Samovski et al., 2023). FAs play a crucial role in the storage and transport of both cholesterol and retinol by forming esters with them (Chang et al., 2006; O’Byrne and Blaner, 2013). FAs can modify proteins, thereby enabling the precise regulation of cellular processes such as intracellular trafficking, subcellular localization, and protein interactions (Jiang et al., 2018). Furthermore, within cells, FAs are stored as neutral lipids, primarily as TAGs and sterol esters, within the organelle lipid droplets (LDs), serving as energy reserves during times of abundance (Walther et al., 2017). LDs were traditionally considered inert cytoplasmic inclusions of fat, but recent research has unveiled their active involvement in maintaining cellular lipid and energy balance (Xu et al., 2018). Therefore, achieving precise delivery of fatty acids and studying the resulting changes in LDs are crucial for understanding the cellular and physiological effects of FAs, as well as unraveling the functions of LDs in lipid and energy homeostasis. To accomplish this, it is critical to prepare a stable and precisely controlled FA stock solution to ensure that FAs are used quantitatively and their effects are reproducible in research, avoiding both overloading and insufficient loading.

Here, we provide a method to efficiently prepare FA stock solution that is reproducible and steady and can be used in a wide range of studies *in vivo* and *in vitro*.

Due to amphipathic nature of FAs, in our method, ultrasonication is applied to improve the solubility and bioavailability of FAs, potentially leading to the formation of FA micelles. We select oleic acid (OA) and palmitic acid (PA), the two most abundant FAs in mammals, to serve as representatives of a typical long-chain monounsaturated FA and a long-chain saturated FA, respectively. Crucially, sodium oleate is utilized for OA and sodium palmitate for PA, as sodium salts of FAs exhibit increased polarity and, as a result, can readily form micelles compared to their non-sodium counterparts. Furthermore, ethanol is selected to augment the solubility and stability of FA micelles. Ethanol is favored due to its two-carbon chain, which readily integrates into the hydrophilic end of FAs, effectively stabilizing the micelles formed by FAs. Notably, ethanol is preferred over other alcohols due to its lower toxicity, despite the fact that alcohols with longer carbon chains may offer more robust integration into FA micelles.

Briefly, FAs are added into ethanol to a desired concentration (Fig. 1A). Then, the mixture is sonicated on ice until it transforms into a milky solution (key stage) (Fig. 2A). Prepared FA stock solution is kept at 4°C and protected from light. The stock solution can be introduced into a culture medium or buffer without albumin to load FAs at the desired concentration (Fig. 2D; Videos S1 and S2). This protocol allows yield of 1 mL 100 mmol/L FA stock solution within 10 min. The FA stock solution should be stocked at 4°C, kept in the dark, and sealed to prevent ethanol evaporation and FA oxidation. It maintains viability for over 6 months. The prepared FA stock solution can be introduced directly into an aqueous working system with or without albumin, for both *in vivo* and *in vitro* studies.

**Figure 1.**
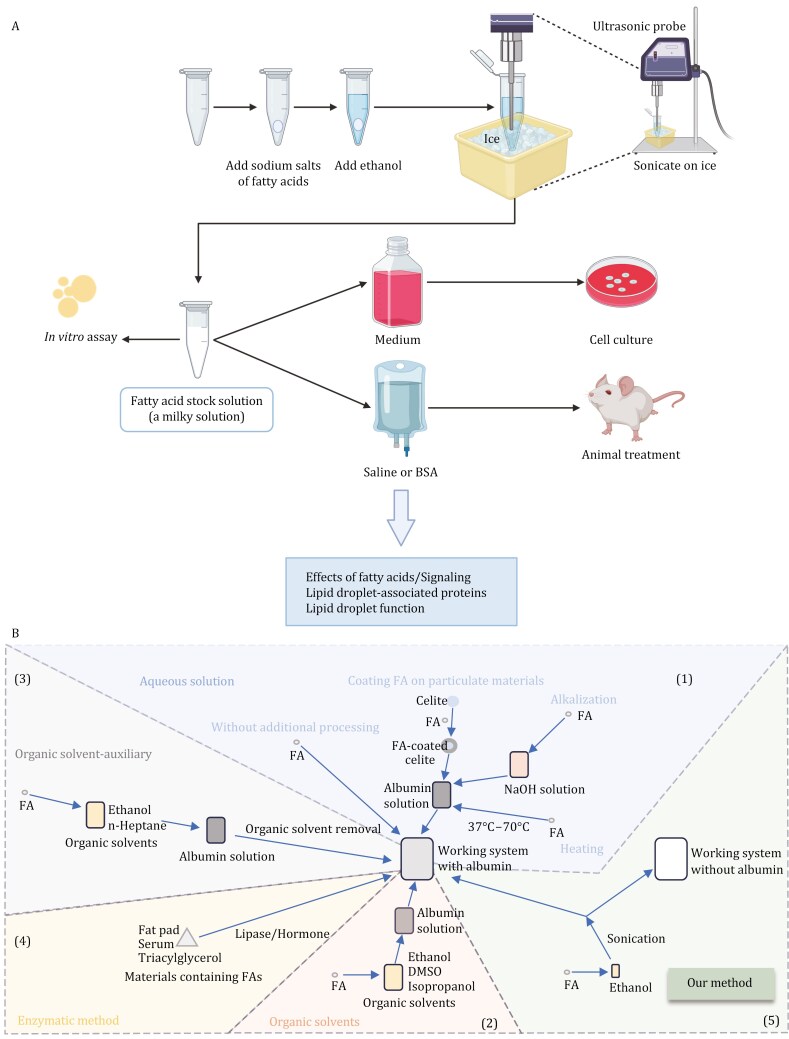
Schematic representation of our method for preparing fatty acid stock solution and comparison with current methods for loading FAs. (A) A simplified flowchart outlining the preparation of an FA stock solution through our method. Briefly, FA sodium salt is quantified in a tube, followed by the addition of ethanol. Then the mixture is sonicated on ice until it appears like a milky solution, that is, FA stock solution, which should be kept at 4°C in the dark. The stock solution can be used directly both *in vivo* and *in vitro*. The figure was created with BioRender.com. (B) A comparison of FA loading methods, with numbering corresponding to Table S1.

**Figure 2.**
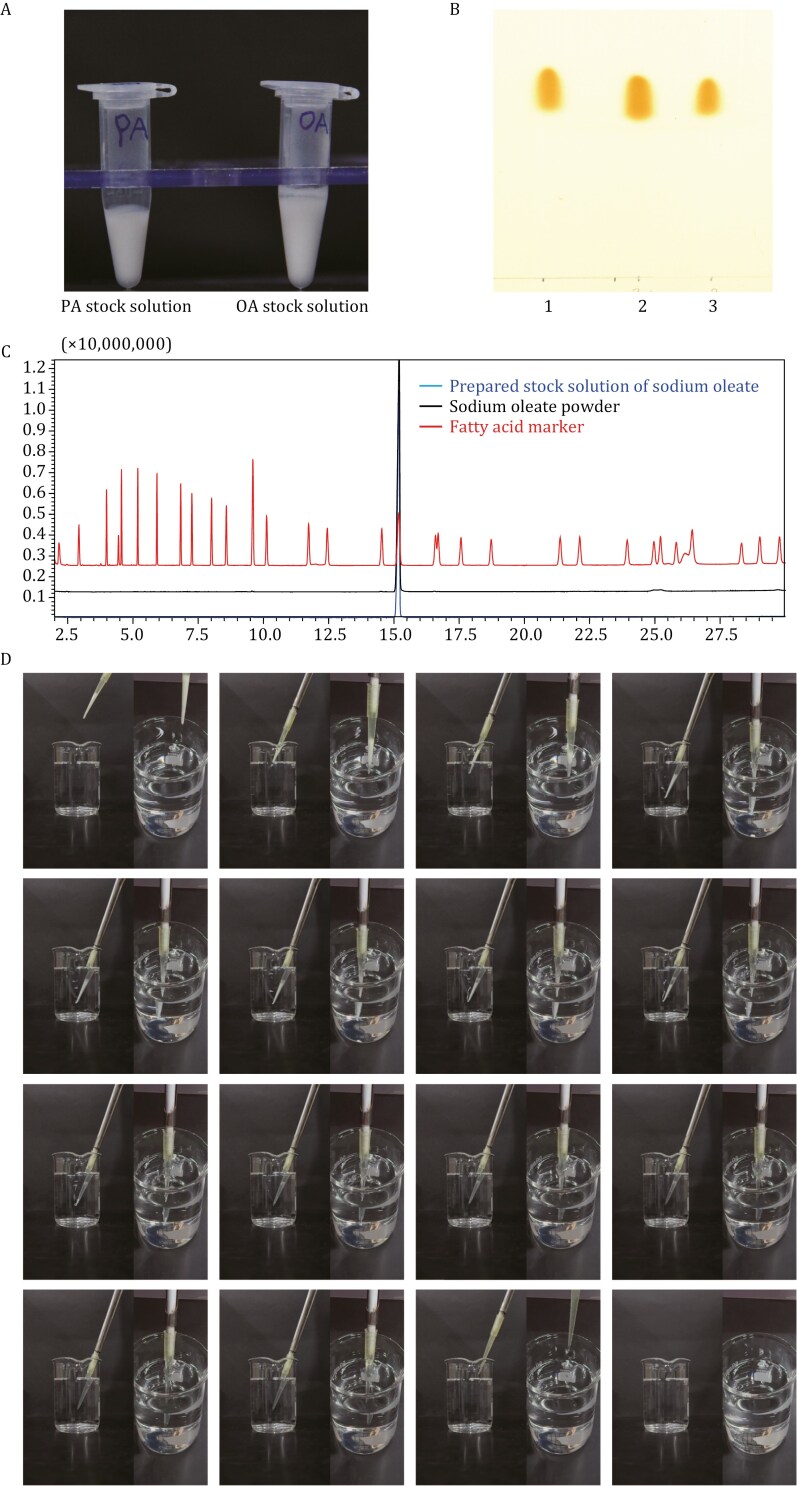
Quality control of the prepared fatty acid stock solution and delivery of OA prepared using this method into water. (A) The milky solution of the prepared FA stock. Left: PA, right: OA. (B and C) Composition analysis of sodium oleate stock solution through (B) TLC, (1) prepared stock solution of sodium oleate; (2) mixture of sodium oleate in chloroform and the prepared stock solution; (3) sodium oleate in chloroform, and (C) GC-MS result. (D) 50 μL of 100 mmol/L OA prepared through this method was added into 50 mL water at room temperature. The entire process has been recorded and supplemented with Video S2. Here are some selected representative shots for reference.

We have summarized and compared the existing methods to deliver FAs in Fig. 1B, Table S1, and Fig. S1. Our technique is both distinct and unique. A flow diagram of the workflow is shown in Fig. 1A. The utilization of our technique offers several key benefits and significant advancements:

(1) The method is both convenient and time-saving. It relies solely on sonication to enhance the solubility of FAs, eliminating the need for heating or additional albumin. Typically, the entire preparation process can be completed in just a few minutes.
(2) The method eliminates the need for albumin to form an albumin-FA complex for delivery of FAs, thus avoiding the introduction of extra albumin into the working system. Moreover, extra albumin in the working system is unnecessary when utilizing our prepared FAs. This helps prevent potential effects, such as inflammation and the quenching of endogenous lipids, associated with albumin (Alsabeeh et al., 2018).
(3) During preparation, no effect on FAs is observed. The results from thin-layer chromatography (TLC) (Fig. 2B) and gas chromatography-mass spectrometry (GC-MS) (Fig. 2C) indicate that there is no difference in composition between the stock solution and the original sodium oleate powder, suggesting that the molecular structures of FAs remain unaffected during the preparation process.
(4) Using this method, a stock solution of FAs with a known concentration can be readily prepared. More importantly, a precise delivery of FAs at a known concentration can be achieved. Therefore, this approach consistently yields reproducible effects when loading FAs, even across independent studies, thus highlighting this valuable advantage. Moreover, with this protocol, the FA stock solution is added immediately before use, preventing the potential loss of free FAs (FFAs) that might occur if FFA-BSA were precomplexed during preparation, possibly due to aggregation (Oliveira et al., 2015).
(5) Researchers can achieve a wide range of FA concentrations using this method, allowing for the investigation of FA functions on various biological processes and systems.
(6) Furthermore, the protocol can be applied to saturated, monounsaturated, and polyunsaturated FAs. For instance, in addition to PA and OA, our method has also proven successful in preparing linoleic acid (LA) and stearic acid (SA) for studying insulin signaling (Fig. S2). This broad spectrum of applicable FAs makes the method appealing to a wide range of users, especially those looking to compare the effects of different FAs or embark on projects involving unfamiliar FAs.
(7) The prepared FA stock solution can be utilized for conducting both *in vivo* and *in vitro* studies, especially for research related to LDs and signaling, making a meaningful contribution to the knowledge and advancement of research in this field.

Therefore, our approach would be of interest to researchers studying FA signaling, membrane formation, lipid metabolism, LD dynamics, metabolic diseases, and beyond. Actually, we have provided our FA stock solution to more than 10 labs since 2008 and the prepared FA solution has been successfully employed in investigating the functions of FAs, the function and dynamics of LDs, and the regulation of lipid metabolism, both *in vivo* and *in vitro*, as detailed below.

First, the delivery of FAs through the prepared solution has yielded dependable and replicable outcomes in various independent studies investigating FA functions, such as the distinct effects of saturated and unsaturated FAs on cell signaling, LD dynamics, and lipid metabolism (Table S1). For instance, specific concentrations of OA and PA have been administered to myoblasts and a system to study their effects on LD size, lipid distribution, ER stress, and insulin resistance has been established. Several independent studies utilizing the prepared FA solutions have consistently revealed distinct effects on LD size between PA and OA. Besides, it has been observed that PA can trigger ER stress and insulin resistance while OA can counteract the effects induced by PA (Figs. S3 and S4).

Moreover, analyzing the changes in the protein profile of LDs after the application of the prepared OA could unveil vital and new proteins crucial in LD dynamics and the regulation of lipid metabolism. For instance, when integrated with proteomics, imaging, and biochemical techniques, the application of the preparation has facilitated the identification of LD-associated proteins that play essential roles in lipid regulation, such as CGI-58, COX-2, and LD-associated noncoding RNA-encoded proteins (LDANP1 and LDANP2). CGI-58, a known activator of lipolysis, was initially observed to exhibit an increased accumulation on LDs following the treatment of CHO K2 cells with our OA solution (Fig. S5A) (Liu et al., 2004). Also, comparative proteomic study of myoblasts treated with the prepared FAs reveals the participation of COX-2 in the development of insulin resistance induced by PA. Similarly, through the analysis of LD proteins subsequent to the treatment of cells with our OA, two novel proteins, LDANP1 and LDANP2, coded by nominally noncoding transcripts, were identified on LDs (Fig. S5B and S5C). The investigations have shown that LDANP1 is engaged in regulating the storage of neutral lipids and insulin signaling processes within myoblasts.

Furthermore, using the prepared OA, we successfully developed a novel *in vitro* assay for examining FA incorporation by isolated LDs to investigate LD-mediated lipid synthesis, shedding light on the role of LDs in the synthesis of phospholipids and TAG. The results show the isolated LDs nearly depleted of ER have an inherent capacity for lipid synthesis when provided with Coenzyme A (CoA), ATP, and OA, suggesting LD is a site for cellular lipid synthesis (Fig. S6). This assay would enable researchers to investigate the specific role of LDs *in vitro* directly.

In the Supplemental Material, we provide detailed procedures for our method of preparing FA solutions and its successful applications, along with guidance to troubleshoot issues. These procedures ensure precise delivery and utilization of FAs and are critical for understanding their cellular and physiological effects, enhancing the study of LDs, and elucidating the underlying mechanisms in lipid metabolism.

As described above, beyond the reliable and significant findings achieved using our FA solution, our method stands out from other preparation and loading techniques by offering the flexibility to introduce FAs into solutions either with or without albumin. This led us to investigate whether the presence or absence of albumin in the system would impact FA uptake by cells (Fig. S1).

In brief, cells were cultured in three media: 10% FBS (standard culture medium), 0.4% BSA (typical albumin levels of 0.2%–0.5%), and albumin-free medium (Hassain et al., 1997), and then treated with 100 µmol/L OA containing 2 µCi/mL [^3^H]-OA. OA incorporation was quantified, showing time-dependent uptake of [^3^H]-OA in HEK293A cells across all conditions, reaching saturation, consistent with previous findings (Samovski et al., 2023). Notably, cells in albumin-free medium had higher OA uptake than those in 0.4% BSA, suggesting albumin slows OA incorporation. This likely results from FAs needing to dissociate from albumin before transport (Samovski et al., 2023; van der Vusse, 2009). These results highlight the impact of albumin concentration on FA incorporation rates (Abumrad et al., 1998). Similar results in Huh7 cells underscored albumin’s impact on FA uptake rates and differences between cell lines. In addition, precise measurement of BSA in BSA-complexed FAs is challenging, complicating control setups with consistent BSA levels. Our albumin-free FA preparation method enables accurate FA quantification without introducing extra albumin that could affect FA effects, ensuring reproducible results.

In summary, an FA stock solution is prepared by sonicating FA salts in ethanol, a simple, 10-min process requiring no specialized expertise. This method avoids the need for albumin, eliminating extra albumin in the system. The resulting homogeneous milky solution can be stored for at least 6 months and directly used in culture medium or water/saline. It accommodates various FAs, including saturated, monounsaturated, and polyunsaturated types. This approach ensures precise FA concentration and consistent research results, supporting studies in FA binding, lipid regulation, LD research, and *in vitro* assays.

## Supplementary Material

pwae068_suppl_Supplementary_Video_S1

pwae068_suppl_Supplementary_Video_S2

pwae068_suppl_Supplementary_Material

## Footnotes

This work was supported by the National Key R&D Program of China (Grant Nos. 2023YFA1801103, 2018YFA0800700, and 2018YFA0800900) and National Natural Science Foundation of China (Grant Nos. 92357302, 32170787, 91954108, and 32100557). The original data that support the findings of this study are available from the corresponding author upon request. The authors declare that they have no competing interests. P.L. and S.Z. conceived and designed the project. M.Z., S.X., S.Z., X.F., Q.L., B.P., and L.C. performed the experiments. S.Z., P.L., M.Z., and S.X. wrote the manuscript. All authors have read and approved the manuscript.
