# Supplementary material for: Preparation of fatty acid solutions for investigating lipid signaling, metabolism, and lipid droplets
Source: Protein Cell. 2024 Dec 17;16(7):609–14. doi: 10.1093/procel/pwae068 (PMC12275089; doi:10.1093/procel/pwae068)
Supplement: pwae068_suppl_Supplementary_Material [file pwae068_suppl_supplementary_material.pdf]

Preparation of fatty acid solutions for investigating lipid signaling, metabolism, and lipid droplets

Shuyan Zhang[1,2,3,4,5,#,*], Mengwei Zhang[5,6,7,#], Shimeng Xu[5,#], Xiaochuan Fu[5], Qiumin Liao[1,2,3,4], Bin Pan[5], Liujuan Cui[5], Pingsheng Liu[5,7,*]

[1]National Key Laboratory of Intelligent Tracking and Forecasting for Infectious Diseases, Beijing Ditan Hospital, Capital Medical University, Beijing 100015, China

[2]Beijing Key Laboratory of Emerging Infectious Diseases, Institute of Infectious Diseases, Beijing Ditan Hospital, Capital Medical University, Beijing 100015, China

[3]Beijing Institute of Infectious Diseases, Beijing 100015, China

[4]National Center for Infectious Diseases, Beijing Ditan Hospital, Capital Medical University, Beijing 100015, China

[5]Institute of Biophysics, Chinese Academy of Sciences, Beijing 100101, China

[6]College of Life Science and Technology, Huazhong University of Science and Technology, Wuhan 430074, China

[7]University of Chinese Academy of Sciences, Beijing 100049, China

[#]These authors contributed equally.

*To whom correspondence should be addressed. E-mail: syzhang@ibp.ac.cn, pliu@ibp.ac.cn

Supplemental Table 1 Summarization of the methods used for delivery of fatty acids.

Methods/Solvents	Brief strategies or Examples	Limitations	References
**(1) Adding FAs in aqueous solution directly**			
**Method 1**: Adding FAs in aqueous solution without extra treatment	Add FAs into medium or other aqueous solution directly prior to use.	● Low solubility, especially for long-chain FAs.	[1-3]
**Method 2**: Dissolving FAs in aqueous solution by coating FA on particulate materials and then complexing FA with albumin	Use particulate materials to increase the contact surface areas between FAs and aqueous solution.<br>Example: "Celite Method"<br>1) Coat the FA in hexane on a particulate material (such as celite);<br>2) Evaporate the organic solvent under nitrogen;<br>3) Incubate the FA-coated celite with albumin solution to form a complex with the FAs and albumin, then remove the celite;<br>4) Dilute the FA-albumin solution with fatty acid-free albumin solution.	● The precise concentration of FA remains uncertain.<br>● The particles could potentially bind to and consequently extract FA from the aqueous solution.<br>● Albumin is required for the preparation of the FA solution.<br>● The process is intricate and time-consuming.	[4]
**Method 3**: Dissolving FAs in aqueous solution facilitated by alkalization and then complexing FA with albumin	1) Dissolve FA sodium salts in a solution of NaOH, or incubate FAs with NaOH to convert them to sodium salts through gentle warming with NaOH solution;<br>2) Combine the resulting solution with albumin to form a complex between the FAs and albumin.	● Low solubility in NaOH solution.<br>● Alkalization of FAs introduces uncertainty regarding the precise concentration, and also excessive heat or exposure to air can induce oxidative damage.<br>● Albumin is indispensable for the preparation of the FA solution.<br>● The process is intricate and	[5-7]

		time-consuming.	
**Method 4**: Heating to promote the solubility of the FAs and then complexing FA with albumin	1) The heating temperatures range from 37°C to 70°C across different protocols.<br>2) Combine the resulting solution with albumin to form a complex of fatty acids with albumin.	● Low solubility in solution persists even with heating.<br>● Albumin is indispensable for the preparation of the FA solution.	[8-10]
**(2) Using organic solvents to prepare FA stock solution**			
Ethanol/DMSO	The protocols usually contain two steps:<br>1) Dissolve FAs in ethanol or DMSO directly or by heating to prepare stock solution;<br>2) Add stock solution into albumin solution/media, sometimes in combination with heating, vortexing, sonicating or shaking.	● Albumin is indispensable for the preparation of the FA solution.<br>● Achieving a high concentration in ethanol is challenging, often requiring heating, which could potentially damage FAs.	[3, 5, 11-23]
Isopropanol	Dissolve FAs in isopropanol directly.	● The stock solution of FA is consistently below 40 mM due to its low solubility in isopropanol.<br>● For use, additional albumin in culture media is always required.	[24, 25]
**(3) Using organic solvents to facilitate the dissolution of FAs and subsequently eliminating the organic solvents at the conclusion of the process**			
Ethanol	1) Dissolve sodium palmitate in 95% ethanol at 60°C to yield stock solution;<br>2) Remove ethanol in palmitate stock solution by nitrogen gas;<br>3) Conjugate palmitate with FA-free BSA.	● Albumin is indispensable for the preparation of the FA solution.<br>● The process is intricate and time-consuming.	[26]

n-heptane	1) Incubate albumin solution with an n-heptane solution containing FA;<br>2) Remove the hydrocarbon phase in the end.	● Fully removing all of the organic solvent presents a challenge.	[27]
Hexane	1) Dissolve FA in hexane and place in a beaker;<br>2) Add NaOH/KOH/NH4OH;<br>3) Evaporate the solvent under N_2;<br>4) Dissolve the FA salt in water (often producing a soap film at the surface);<br>5) Add dropwise to the albumin solution;<br>6) Adjust pH and dilute with water;<br>7) Measure FFA concentration;<br>8) Dilute with FA-free albumin solution.		[4]
**(4) Enzymatic method**			
	Incubate materials containing FAs (such as fat pad, serum, TAG) with lipase or hormone in albumin solution.	● Final solutions of FAs typically constitute a mixture, dependent on the materials utilized, making it challenging to control the types and ratios of the FAs obtained.<br>● The precise concentration of FA remains uncertain.<br>● The process is intricate and time-consuming.	[27]
**(5) Sonication FAs in ethanol (our protocol)**			
	Sonicate the FA salt in ethanol.		[28-39]

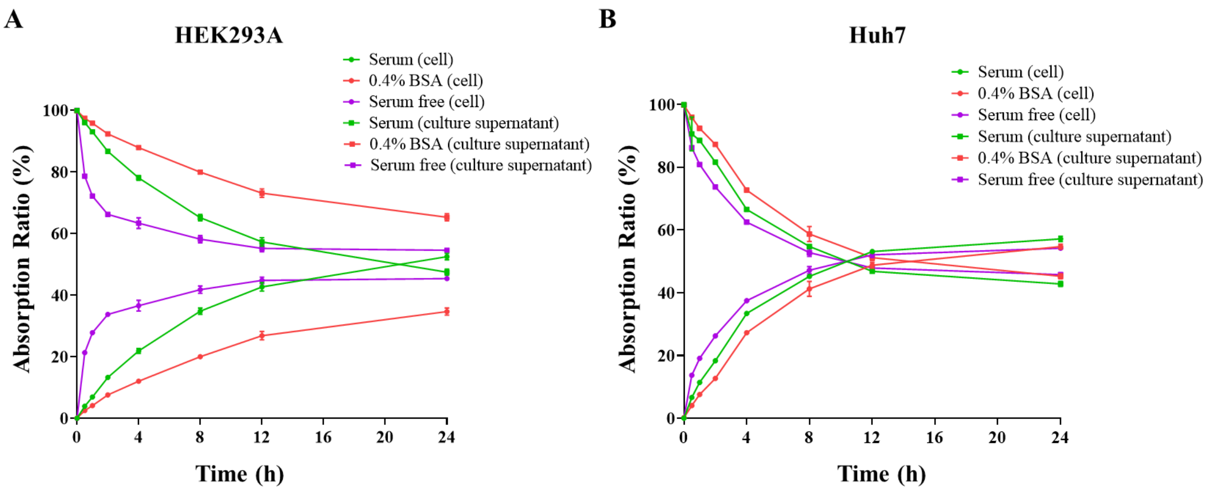

Supplemental Figure 1 Effects of albumin on the incorporation of radio-labeled OA into cells.

In brief, cells were initially grown in standard DMEM medium with 10% FBS until they reached 100% confluence. Following this, the cells were exposed to different DMEM culture media: one with 10% FBS, another with 0.4% BSA, and one with no albumin. Subsequently, the cells were exposed to a 100 µM oleate solution containing 2 µCi/mL of [^{3}H]-oleate for varying time intervals. At specified time points, the culture medium was collected, and the cells were harvested after two washes with PBS. The cell lysates were prepared using saline containing 1% SDS. The radioactivities in media and cell lysates were measured using a PeckinElmer scintillation counter.

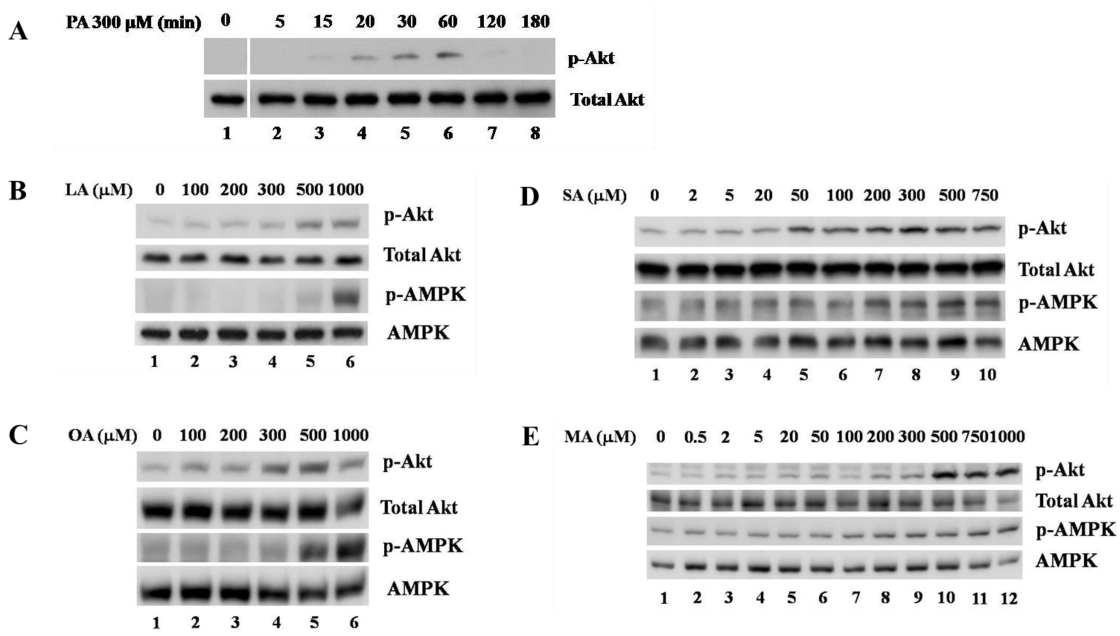

Supplemental Figure 2 The prepared saturated, monounsaturated and polyunsaturated fatty acids affect insulin signaling in a dose- and time-dependent manner.

A. L6 myotubes were exposed to 300 µM PA for the specified durations, and p-Akt was assessed using Western blotting. (**B-E**). L6 myoblasts were exposed to different FAs, namely linoleic acid (LA) (**B**), oleic acid (OA) (**C**), stearic acid (SA) (**D**), and a combination of PA, LA, OA, and SA (at a ratio of 2.77:2.56:1.77:1, representing a mimic of FA composition akin to that in rat plasma, MA) (**E**), each at the specified concentrations for 30 minutes, and cell lysates were collected and subjected to Western blotting. Figures are modified with permission from reference [33].

Supplemental Video 1-FA into DMEM containing FBS

Supplemental Video 2-FA into water

Detailed protocol and critical procedures step-by-step for preparing FA solutions and their applications

Reagents

Sodium oleate (Sigma-Aldrich, cat. no. O7501)

Sodium palmitate (Sigma-Aldrich, cat. no. P9767)

Ethanol (Sinopharm, cat. no. 10009218)

Chloroform (Sinopharm, cat. no. 10006818) **! CAUTION** Highly toxic and carcinogenic, chloroform can harm the liver, and induce dizziness, headaches, and nausea. Use it only in a fume hood, protect yourself with gloves, lab coat, and safety glasses.

Diethyl ether (Sinopharm, cat. no. 10009318) **! CAUTION** Highly flammable and capable of causing dizziness, headaches, and nausea, diethyl ether demands careful handling in a fume hood while wearing gloves, lab coat, and safety glasses.

Acetic acid (Sinopharm, cat. no. 10000218) **! CAUTION** This corrosive substance can cause skin burns and eye damage. Employ gloves, lab coat, and safety glasses for protection.

Hexane (Sinopharm, cat. no. 80068662) **! CAUTION** This flammable and irritant liquid can irritate skin and eyes. Wear gloves, lab coat, and safety glasses, and work in a well-ventilated area.

Methanol (Sinopharm, cat. no. 80080418) **! CAUTION** This flammable and toxic liquid can lead to blindness and damage the central nervous system. Use it only in a fume hood while wearing gloves, lab coat, and safety glasses.

Acetone (Sinopharm, cat. no. 1000418) **! CAUTION** Acetone is highly flammable and can be explosive under certain conditions. Avoid flames and handle it in a fume

hood.

Free fatty acid-free BSA (Sigma-Aldrich, cat. no. SRE0098)

LipidTOX Red (Thermo Fisher Scientific, cat. no. H34476)

Hoechst 33342 (Thermo Fisher Scientific, cat. no. 62249)

Paraformaldehyde (Electron Microscopy Sciences, cat. no. 1578) **! CAUTION** This carcinogenic and irritant substance can irritate the respiratory system and skin. Use it in a fume hood while wearing gloves, lab coat, and safety glasses.

Percoll (Sigma-Aldrich, cat. no. 17-0891-01)

LR White resin (Electron Microscopy Sciences, cat. no. 14380)

Uranyl acetate (Electron Microscopy Sciences, cat. no. 22400) **! CAUTION** This radioactive and toxic substance can cause kidney damage. Handle it with extreme care, dispose of it properly, and follow all safety guidelines for radioactive materials.

KRBH buffer (see ‘Reagent setup’)

Buffer A (see ‘Reagent setup’)

Buffer B (see ‘Reagent setup’)

2 × SDS sample buffer (see ‘Reagent setup’)

ATP (Sigma-Aldrich, cat. no. A7699) **! CAUTION** Prepare freshly

CoA (Sigma-Aldrich, cat. no. C4282)

[^{3}H] Oleic acid (PerkinElmer Life Sciences, cat. no. NET289005MC) **! CAUTION** This radioactive material can lead to internal radiation exposure. Handle it with extreme care, dispose of it properly, and adhere to all safety guidelines for radioactive materials.

[^{3}H] Palmitic acid (PerkinElmer Life Sciences, cat. no. NET043001MC) **! CAUTION** This radioactive material can lead to internal radiation exposure. Handle it with extreme care, dispose of it properly, and adhere to all safety guidelines for radioactive

materials.

Equipment

Analytical balance (Sartorius, model no. BS124S)

Ultrasonic homogenizer (Cole Palmer, model no. CPX 600; Scientz-IID, Tip, $\phi 2$, 2 mm)

GC-MS (Shimadzu, model no. GCMS-QP2010)

Confocal microscope (Olympus, model no. FV1200)

Centrifuge (Eppendorf, model no. 5424R)

Centrifuge (Beckman Coulter, model no. Ti70)

CO_2 incubator (Thermo Scientific, model no. 371)

Transmission electron microscope (FEI, model no. Tecnai Spirit)

Scintillation counter (PerkinElmer, model no. 1450 MicroBeta TriLux)

Reagent setup

KRBH buffer: Krebs-Ringer HEPES buffer, 120 mM NaCl, 25 mM HEPES, 4.6 mM KCl, 1 mM $MgSO_4$, 1.2 mM KH_2PO_4 , and 1.9 mM $CaCl_2$, pH 7.4)

Buffer A (25 mM tricine, 250 mM sucrose, pH 7.8)

Buffer B (20 mM HEPES, 100 mM KCl, and 2 mM $MgCl_2$, pH 7.4)

2 × SDS sample buffer: 100 mM Tris-HCl (pH 6.8), 4% SDS (m/v), 20% glycerol (v/v), 4% 2-mercaptoethanol (v/v, added freshly before use)

Procedure

Before starting ● TIMING ~ 4 min

▲ CRITICAL Selecting the sodium salt of a FA over the FA itself is preferable because the sodium salt can more readily form micelles. Additionally, ethanol should be used in the preparation process as it enhances the formation of micelles

and has lower toxicity.

1) To prepare 1 mL of 100 mM stock solution of FA, place 0.030 g sodium oleate or 0.028 g sodium palmitate into a 1.5 mL Eppendorf tube.
2) Add around 1 mL of ethanol into the tube.
3) Place the Eppendorf tube on ice for 1 min.

Ultrasonication ● TIMING ~ 5 min

4) Sonicate the aforementioned mixture on ice at 70 W, with 10-second intervals on and 3-second intervals off, repeated six times. During this process, gently move the tube up and down to ensure that all visible FA precipitates are sonicated. Continue sonication until the solution becomes milky and homogeneous (Fig. 2A).

▲ **CRITICAL STEP** Sonication should always be conducted on ice. It is essential to carefully adjust and monitor both the intensity and duration of sonication to prevent the generation of heat, which can potentially damage the FAs.

? TROUBLESHOOTING

5) Examine the prepared stock solution under light to ensure that all precipitation has disappeared, which is a critical indicator of successful preparation.

▲ **CRITICAL STEP** The milky solution should be free of any visible particles at the end of the process.

? TROUBLESHOOTING

Assessment of the quality ● TIMING Variable

6) Analyze the composition of the prepared FA stock solution using both TLC (Fig. 2B) and GC-MS (Fig. 2C).

(A) TLC analysis ● TIMING ~ 1 h

(i) Dissolve the powders of FA salt in chloroform as a control.

(ii) Apply the prepared stock solution and the control sample onto a silica gel plate.

(iii) Develop the plate in a solvent system of hexane/diethyl ether/acetic acid (80:20:1, v/v/v).

(iv) Visualize the plate by exposing it to iodine vapor for unsaturated FAs or sulfuric acid for saturated FAs.

(B) GC-MS analysis ● **TIMING** ~ Variable

(i) Dissolve the powders of FA salt or dilute the prepared FA stock solution in deionized water using a water bath set at 60°C.

(ii) Acidify the sample to pH 2 by adding 3 M sulfuric acid. Upon cooling to room temperature, the fatty acid will float on the surface.

(iii) Collect the fatty acid onto an organic membrane filter using vacuum filtration, and rinse the filter three times with deionized water.

(iv) Wash the organic membrane filter with hexane and collect the hexane, then evaporate the hexane under a steam of N_2.

(v) Dissolve the resulting sample in 200 μL of hexane.

(vi) Prepare the esterification reagent by mixing 1 mL of a solution containing methanol and concentrated sulfuric acid in a 39:1 (v/v) ratio.

(vii) Add 10 μL sample in step (v) into the esterification reagent and keep the reaction in 70°C water bath for 1~2 h. Then cool the reaction at room temperature for 5~10 min.

(viii) Introduce 1.5 mL of deionized water and 200 μL of hexane into the reaction system and vortex the tube vigorously.

(ix) Centrifuge the tube at 3,000*g* for 2 min and collect the upper layer of the solution for GC-MS analysis.

! **CAUTION** Adhere to the worksheet and follow safety instructions diligently when working with toxic and organic solvents.

Storage ● TIMING Variable

7) Securely seal and encase the tube containing the prepared FA stock solution at 4°C.

! **CAUTION** Shield the FA stock solution from light exposure to prevent oxidation.

Usage ● TIMING Variable

8) Properly stored FA stock solutions remain viable for more than six months.

▲ **CRITICAL STEP** In practice, if the OA and PA solutions are stored at 4°C, shielded from light, and tightly sealed, they can actually remain viable for more than six months. Additionally, our methodology is applicable to polyunsaturated FAs. To prolong shelf life, especially for polyunsaturated FAs, it is advisable to flush the tube with nitrogen after each use to prevent oxidation.

9) Before usage, warm the tube to room temperature and gently vortex the solution.

▲ **CRITICAL STEP** Under 4°C, the FA solution may stratify into different concentration layers. Therefore, it is important to allow the tube to equilibrate to room temperature and gently vortex the solution before use to ensure homogeneity. Filtration of the solution before use is unnecessary, as the stock solution is prepared with pure ethanol, ensuring sterility. It is recommended that handling of the stock solutions be conducted within a clean bench to maintain sterility, especially for cell culture experiments.

10) Introduce the desired volume of stock solution directly into experimental solution with or without serum/albumin.

▲ **CRITICAL STEP** After withdrawing the FA solution from the tube, it is crucial to remove any excess solution clinging to the external surface of the

pipette tip by employing a Kimwipe or lint-free wipe. This is important to ensure the precise loading of FAs.

▲ **CRITICAL** As described above, we measure a specific amount of FA salt and then subject it to sonication in ethanol. This process results in a homogenous milky solution, with no residual FA salt left behind, thereby producing a FA stock solution with a known concentration. Furthermore, to verify whether the prepared FAs can be delivered accurately at the desired concentration, we thoroughly documented the dispersion process of the prepared FAs in working systems (Supplemental Video 1, standard medium: DMEM+10% FBS; Supplemental Video 2, water; Fig. 2D). The video recording clearly demonstrates that the loaded FAs disperse immediately into aqueous phase without forming any clots, flocculants, or precipitants. After releasing the FA solution from the pipette, any remaining solution adhering to the pipette tip's walls can be easily pipetted out by repeating the pipetting process several times. Importantly, the solution remains as clear as it was originally. Therefore, our method offers a dependable approach to guaranteeing the accurate delivery of FAs at the desired concentration, even within a system lacking albumin.

Downstream applications and detailed protocols

▲ **CRITICAL** The FA solutions can be applied in a wide range of studies, particularly for investigating signaling, metabolism, and LDs both *in vivo* and *in vitro*. Given that free FA concentrations in the plasma typically range in the hundreds of micromolar range, concentrations of 100 to 500 μM FAs have been employed. Besides, the studies are conducted using the two primary FAs in serum, PA and OA.

Fatty acid treatment protocol for cell culture ● **TIMING** Variable

11) Prior to use, heat the cell culture medium to 60°C for both the FA treatment solution and the control solution.

12) Add the FA stock solution to the preheated cell culture medium containing 10% FBS to achieve the desired final concentration of FA.

    ? TROUBLESHOOTING

13) For the control group, add an equivalent amount of ethanol to the preheated cell culture medium containing 10% FBS.

14) Allow both the FA treatment solution and the control solution to cool to 37°C before using them for cell culture.

15) Incubate the cells in the respective FA-containing medium or control medium for the desired duration.

▲ **CRITICAL STEP** When utilizing OA for cell treatment, there is no need to heat the medium to 60°C. OA treatment solution can be prepared at 37°C and room temperature. However, when working with PA, it is essential to add the stock solution to medium at 60°C to prepare the PA treatment solution and ensure that the solution is cooled to 37°C before adding it to the cells. Moreover, as is well-known, the solubility of FAs in water decreases with longer hydrocarbon chains and fewer double bonds. In this protocol, we employed PA, a saturated FA, and OA, a monounsaturated FA, as examples to illustrate the conditions for using the prepared FA. Typically, a temperature of 37°C is recommended for unsaturated FAs and 55-60°C for saturated FAs when incubating the prepared FAs with culture media, followed by cooling down to 37°C. In fact, adhering to these treatment conditions, the method can successfully prepare not only PA and OA but also LA and SA for studying

insulin signaling, as demonstrated in Supplemental Figure 2.

▲ **CRITICAL** In a physiological context, FA concentrations typically range in the hundreds of micromolar, which serves as a reference for determining the appropriate amount of FA loading. The duration of FA incubation with cells varies according to specific experimental objectives. In general, excessively prolonged treatment times and/or overly high FA concentrations can lead to cellular toxicity.

We have included the treatment durations and FA concentrations in the following sections to investigate the impact of FAs on insulin signaling in skeletal muscle cells. In all, it is critical to explore the appropriate timing and dosage of FA treatment according to the specific goals of the experiment. Furthermore, it is essential to consider the distinct effects of saturated and unsaturated FAs on signaling, LDs, and lipid distribution in cells when selecting and loading FAs.

Influence of fatty acids on insulin signaling in skeletal muscle cells ● **TIMING** Variable

16) Incubate C2C12 cells in the presence or absence of FAs at the specified concentrations and incubation times.
17) After the FA treatment, stimulate the cells with insulin at a concentration of 200 nM for 10 min at 37°C. This step activates the insulin signaling pathway.
18) Following insulin stimulation, immediately lyse the cells directly in 2 × SDS sample buffer to ensure thorough cell lysis.
19) Subject the cell lysate to sonication for breaking down nucleic acids.

▲ **CRITICAL STEP** To prepare protein samples for subsequent analysis, whether obtained from cells or tissues, we recommend adhering to our recently published protocol[40]. The protocol yields high-quality protein samples from

various cell types and nearly all tissue types, addressing concerns related to protein degradation, modification during sample preparation, and potential interference from cellular components like neutral lipids and glycogen. Briefly, the procedure involves sonicating the collected tissue or cells in 2 × SDS sample buffer until complete homogenization is achieved. Subsequently, the protein lysate is subjected to TCA precipitation to eliminate other components such as glycogen, lipids, and nucleic acids.

? TROUBLESHOOTING

20) Conduct Western blotting to analyze the proteins of interest related to insulin signaling, such as p-Akt *et al*.

Here, following a 12-h incubation with 500 µM PA, skeletal muscle cells exhibited a complete loss of Akt phosphorylation in response to insulin. However, a 12-h incubation with 200 µM OA could not affect insulin-induced Akt phosphorylation in skeletal muscle cells. When incubating skeletal muscle cells with both FAs, it is demonstrated that 200 µM OA can restore the Akt phosphorylation response to insulin induced by 500 µM PA (Supplemental Fig. 3A).

Also, acute impact of FAs on insulin signaling in skeletal muscle cells can be studied using the prepared PA solution. Akt activation in L6 myoblasts, induced by 300 µM PA, is observed at 10 min, reaches its peak at 45 min, and becomes undetectable after 3 h (Supplemental Fig. 3C).

Impact of fatty acids on insulin signaling in rat skeletal muscle following infusion

21) Ensure that the rats have undergone jugular vein catheter placement surgery and have had at least 4 days to recover.

22) Randomly divide the rats into four treatment groups: BSA, PA, OA, and PA plus OA.

23) Adapt the rats to 30-min slow saline infusions at a rate of 5 μL/min to keep the infusion lines open.

24) Dissolve PA and OA separately or together in 1% FA-free BSA at 60ºC to achieve final concentrations of 2.5 mM for PA and 1 mM for OA. Cool the solutions to 37°C before use.

25) Infuse the rats continuously with these four treatments for 12 h at a rate of 2 mL/h, followed by a subsequent 12-h period during which they have free access to food and water, and engage in normal activity. This constitutes one complete infusion cycle.

26) Repeat the above infusion cycle for 7 days.

27) Half an hour after the 7-day lipid infusion period, perform insulin infusion at a rate of 2 U/kg body weight over a 10-min period.

28) After anesthesia, promptly extract the soleus muscles and rapidly freeze them using liquid nitrogen.

29) Store the frozen soleus muscles at -80°C until further analysis.

Similar to cell experiments, insulin-stimulated Akt phosphorylation in rat soleus muscle significantly decreased after 1 week of PA infusion, while OA infusion had no significant effect. Simultaneous infusion of both PA and OA completely blocked the inhibitory effect of PA on insulin-stimulated Akt phosphorylation in the soleus muscle (Supplemental Fig. 3B).

Investigation of the impact of cell surface-bound PA on insulin signaling

30) Incubate C2C12 cells with 300 μM PA at 4°C for 1 h, to prevent PA internalization and achieve PA binding to cell surface.

31) Following PA treatment, wash the cells three times with either ice-cold KRBH buffer to eliminate unbound PA or a 0.4% FA-free BSA-containing KRBH to remove bound PA.

32) After washing, add 2 × SDS sample buffer to the cells for cell lysis or incubate the cells in pre-warmed KRBH buffer at 37°C to initiate signal transduction, and culture the cells for the specified durations.

33) As a positive control, treat a separate set of cells with 100 nM insulin in pre-warmed KRBH buffer at 37°C for 20 min.

34) Following the respective treatments, immediately lyse the cells directly in 2 × SDS sample and subject the cell lysate to sonication to further break down cellular components.

35) Perform Western blotting to detect phosphorylated Akt.

Cell surface-bound PA stimulates Akt phosphorylation starts after 5 min, peaks at 10 min, and subsequently declines (Supplemental Fig. 3D).

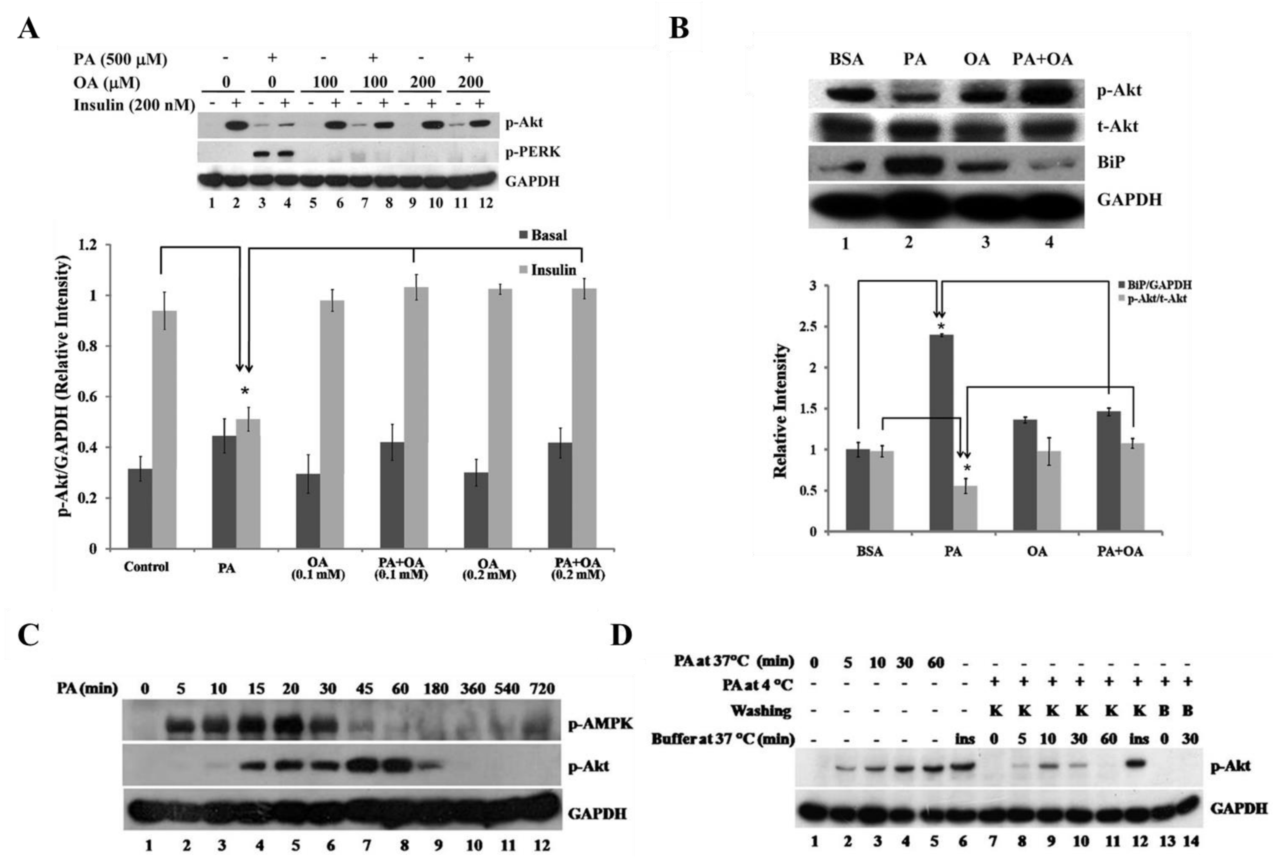

Supplemental Figure 3 Integration on the effects of fatty acids on insulin signaling of skeletal muscle cells.

Consistent effects of the prepared FAs on the insulin signaling of skeletal muscle cells have been observed across independent studies using the prepared FA solutions. **(A)** Insulin sensitivity of C2C12 myoblasts after treatment presence or abcence of 500 μM PA, with or without 100 μM, or 200 μM OA for 12 h before insulin stimulation. **(B)** Insulin sensitivity of rat soleus muscle after treatment with FAs for one week. Sprague Dawley rats were treated with continuous venous infusions with 1% BSA, 2.5 mM PA (in 1% BSA), 1 mM OA (in 1% BSA), and 2.5 mM PA plus 1 mM OA (in 1% BSA) respectively for 1 week. The soleus muscle was collected 10 minutes after insulin injection for analysis. **(C)** L6 myoblasts were exposed to 300 μM PA for brief periods, as indicated and Akt phosphorylation was analyzed using Western blotting. **(D)** Assessment of the impact of cell surface-bound PA on insulin signaling in C2C12

cells. K, KRBH; B, BSA; ins, insulin. (A) and (B) are modified with permission from reference [29]. (C) and (D) are modified with permission from reference [33].

Effects of fatty acids on subcellular lipid distribution in skeletal muscle cells ●

TIMING ~ 6 days

36) Culture C2C12 to confluence in 100-mm dishes.

37) Treat cells with a mixture of 500 μM PA and 0.5 μCi/mL [^{3}H]-labeled PA in the presence or absence of 200 μM OA for 12 h.

38) Rinse cells with cold PBS twice and harvest cells using a cell scraper. After harvesting, centrifuge the cells at 300*g* for 5 min. Homogenize them using a douncer in 2 mL of Buffer A. Collect the supernatant as homogenate after centirfugation at 800*g* for 5 min.

39) Load the homogenate onto a 30% Percoll gradient (10 mL, prepared in Buffer A) in a Beckman Ti70 centrifuge tube.

40) Centrifuge at 80,000*g* for 1 h at 4°C.

41) Carefully collect 1 mL fractions from top to bottom of the gradient into individual 1.5-mL tubes.

42) Perform either Western blotting or total lipid analysis:

(A) Western blotting: Analyze specific proteins according to standard protocols.

(B) Total lipid analysis by TLC

(i) Mix 1 mL lipid extraction reagent (chloroform/methanol/acetic acid, 50:50:1, v/v/v) with 0.5 mL of each fraction and mix thoroughly. Centrifuge the mixture at 10,000*g* for 10 min at 4°C and retrieve the lower portion, which contains the extracted lipids.

(ii) Separate the extracted lipids by TLC using a developing solvent for

neutral lipids (N-hexane/diethyl ether/acetic acid, 80:20:1, v/v/v),.

(iii) Collect the separated lipid fractions from TLC plates.

43) Analyze the collected fractions for radioactivity using a PerkinElmer scintillation counter.

Under the given conditions, OA resulted in the formation of larger LDs compared to PA. PA treatment causes phospholipid accumulation in the ER (with ER expansion), while OA drives lipid conversion to TAG in LDs (Supplemental Fig. 4).

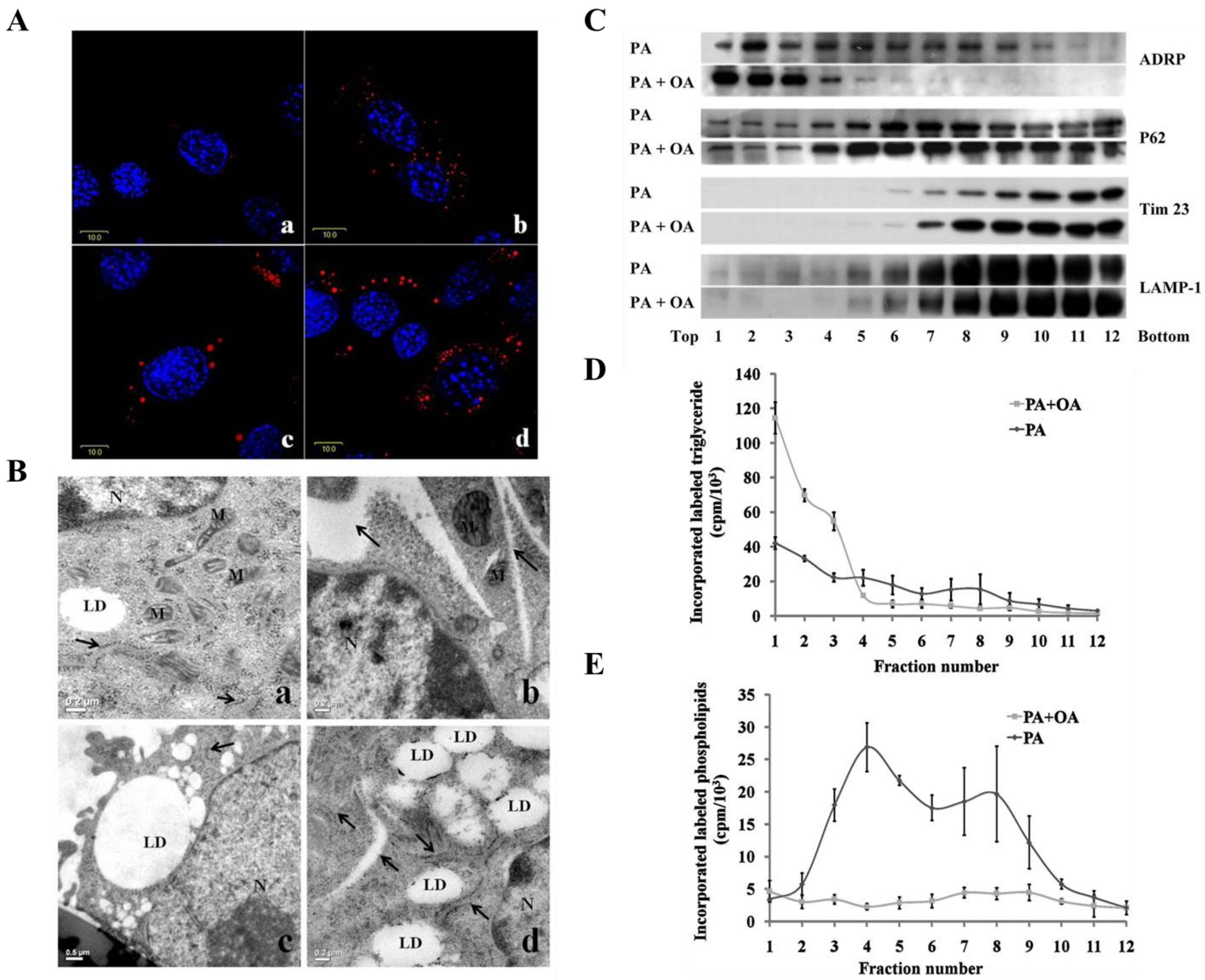

Supplemental Figure 4 The effects of the prepared fatty acid stock solution on LD size and lipid distribution.

(**A**) The size of LDs, revealed by LipidTOX Red staining and viewed by confocal microscopy, in C2C12 myoblasts after treated with a, no FA; b, 500 μM PA; c, 200

μM OA; d, 500 μM PA + 200 μM OA for 12 h. (**B**) ER expansion revealed by transmission electron microscopy after treatment of C2C12 myoblasts with different FAs: no FA, 500 μM PA, 200 μM OA, and 500 μM PA + 200 μM OA for 12 h, respectively. Arrows indicate representative ER structures. N, Nucleus; M, mitochondria; LD, lipid droplets. (**C**) Subcellular distribution of ADRP in C2C12 cells treated with PA and a combination of PA and OA for 12 h. PA, 500 μM PA and 0.5 μCi/mL [^{3}H] PA; OA, 200 μM OA. Subcellular distribution of radiolabeled TAG (**D**) and phospholipids (**E**) in PA and PA plus OA-treated C2C12 cells. (A-E) are modified with permission from reference [29].

Analysis of altered proteins on lipid droplets after fatty acid treatment ●

TIMING Variable

44) Treat cells or experimental animals with the prepared FA.

45) Isolate LDs of high quality.

▲ CRITICAL We strongly recommend following our established and validated protocol, which has been published[41]. Briefly, samples containing LDs are washed and homogenized in a buffer containing 250 mM sucrose to protect intracellular organelles. Then subject the homogenate to ultracentrifugation to separate the LDs from other cellular fractions.

? TROUBLESHOOTING

46) Collect the LD fraction obtained from the ultracentrifugation. Wash the LD fraction several times to eliminate any contaminants.

47) Precipitate the proteins from LD samples using 1 mL of chloroform/acetone (1:1, v/v). Dispose of the organic phase, allow the resulting pellet to air-dry, and subsequently dissolve it in 2 × SDS sample buffer.

48) Separate the LD proteins using SDS-PAGE. In an alternative way, the collected LD proteins in each treatment group can be directly analyzed using proteomics to identify any protein alterations.

49) Visualize protein bands using Colloidal blue staining or silver staining.

50) Identify unique protein bands that appear or disappear, or bands that undergo significant changes following treatment (Supplemental Fig. 5A).

51) Subject the protein bands of interest to proteomic analysis.

52) After selecting proteins of interest from the proteomic data, confirm the changes in protein expression through Western blotting.

▲ **CRITICAL STEP** To discover any changes in proteins after FA treatment, it is essential to ensure that the LD protein profiles remain consistent across various biological experimental replicates.

▲ **CRITICAL** A novel LD-associated protein may be discovered following FA treatment as LDs are active organelles regulating lipid metabolism. When investigating the LD localization of a protein, FA treatment is not mandatory. However, if LDs are too small for detection or isolation, we recommend treating cells with OA rather than PA at an appropriate concentration and duration to enlarge LDs. Additionally, it should be noted that the localization of a protein may vary when FAs are present or absent. For example, Rab18 translocates from the endoplasmic reticulum (ER) to LDs after OA treatment[38].

Localization analysis of a potential LD-associated protein

53) Protein localization and confirmation of its association with lipid droplets should be investigated through multiple experimental approaches.

(A) Fluorescent microscopy. ● **TIMING** ~ 1 day

(i) Cultivate cells expressing the GFP-tagged protein on glass cover slips in

the presence of 100 μM OA for 10 h.

(ii) Rinse cells with cold PBS three times and stain them with LipidTOX Red (1:1,000) for 30 min for lipid droplets, and Hoechst 33342 (1:1,000) for 10 min for nuclei.

(iii) Utilize a confocal microscope to visualize the cells and observe the localization of the GFP-tagged protein. Alternatively, if the protein is not tagged with a fluorescent protein, immunofluorescence staining of the protein should be performed.

The presence of distinct GFP fluorescence in ring-shaped structures surrounding LipidTOX Red-stained LDs indicates its co-localization with the LDs (Supplemental Fig. 5B).

(B) Immuno-gold electron microscopy for more precise localization of the protein.

● TIMING ~ 10 days

(i) Collect the cells by trypsin digestion and fix the cells in 4% (m/v) paraformaldehyde overnight at 4°C.

(ii) Dehydrate the cells in an ascending ethanol concentration series at room temperature: 50%, 70%, and 80%, for 15 min each.

(iii) Incubate the samples in a 2:1 mixture of LR White resin to 70% ethanol for 1 h.

(iv) Perform three changes of 100% LR White resin, with incubation times of 1 h, 12 h, and 1 h, respectively.

(v) Place the cells in LR White resin within a gelatin capsule and polymerize at 50°C for 24 h.

(vi) Prepare 70 nm ultrathin sections of the resin block and mount the ultrathin sections onto a formvar-coated copper grid.

(vii) Incubate the sections on a drop of PBS with 1% BSA and 0.15% glycine for 10 min.

(viii) Transfer the sections to a drop of GFP polyclonal antibody (TaKaRa, Cat. No. 632592) at a 1:50 dilution for 1 h.

(ix) Subsequently, incubate the sections in a second antibody conjugated with 18-nm gold particles (Jackson, Cat. No. 111-215-144) at a 1:100 dilution for 1 h.

(x) Stain the sections with 1% uranyl acetate for 10 min.

(xi) Observe the sections under a transmission electron microscope.

Colloidal gold particles (black dots) surrounding LDs indicates the LD localization of the protein (Supplemental Fig. 5B). Alternatively, other established procedures can also be utilized for immuno-gold labeling, in addition to the method above.

(C) Cell fractionation for biochemical analysis ● **TIMING** ~ 6 days

(i) Harvest the cells for subsequent cell fractionation.

(ii) Fractionate the cells into distinct fractions, including LD, cytosol, total membrane, and post-nuclear supernatant, as detailed in our established LD isolation protocol[41].

(iii) Separate proteins present in various cellular fractions via SDS-PAGE.

(iv) Analyze the separated proteins using Western blotting, employing specific antibodies representing various organelles such as mitochondria and the ER *et al*.

An LD-associated protein should exhibit significant enrichment in the LD fraction compared to the membrane, cytosol, and other fractions. Conversely, marker proteins for other fractions should primarily be detected in their respective fractions

and not in isolated LDs when equal amounts of proteins are loaded (Supplemental Fig. 5B). Protein amounts loaded can be assessed through Colloidal blue staining or silver staining.

▲ **CRITICAL STEP** When assessing the distribution of a protein across cellular fractions, the quality of the LD fraction is crucial.

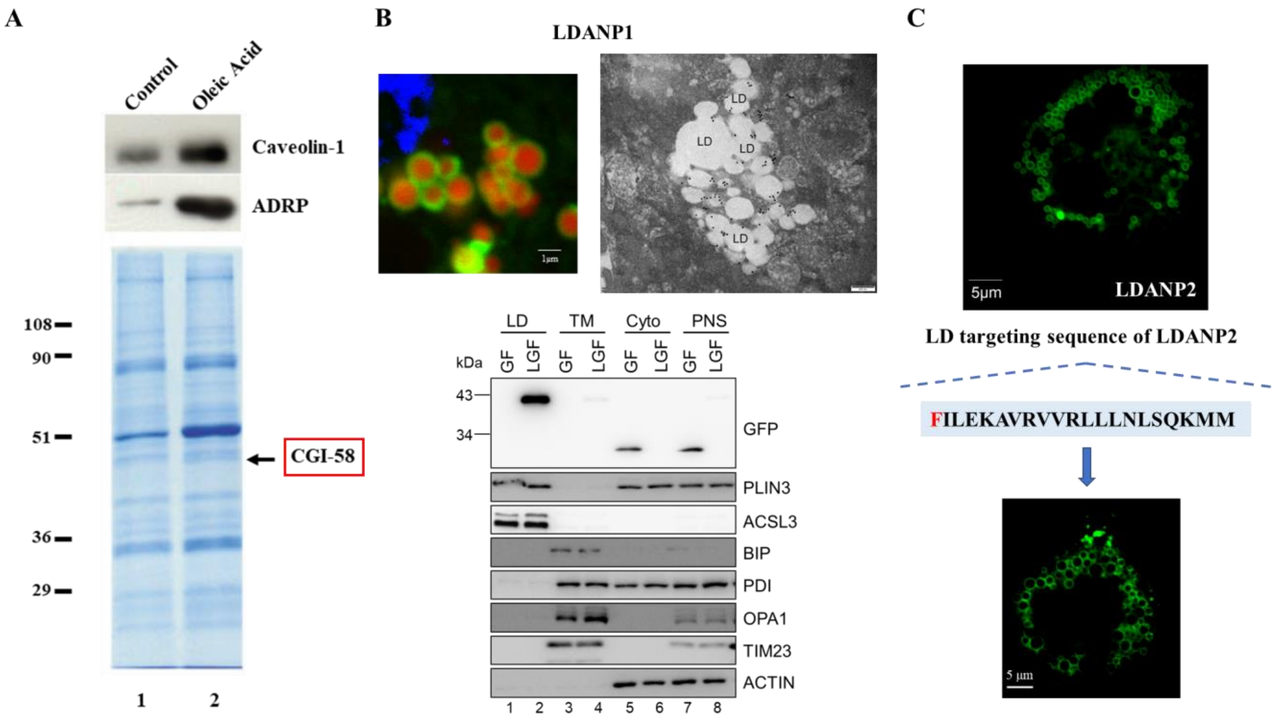

Supplemental Figure 5 The prepared fatty acid solution can be employed to uncover essential proteins involved in LD dynamics and new LD-associated proteins.

(**A**) CGI-58 is revealed to be increased on LDs in the presence of OA. The arrow indicates the band identified as CGI-58 by mass spectrometry. (**B**) and (**C**) The first and second LD-associated noncoding RNA-encoded proteins, LDANP1 and LDANP2, have been discovered after the proteomic analysis of LDs isolated from C2C12 cells treated with the prepared OA. (A), (B) and (C) are modified with permission from references [28], [34] and [35], respectively.

In vitro analysis of fatty acid incorporation into phospholipids and TAG on lipid droplets ● TIMING ~ 3 days

54) Mix 2 μL of [^{3}H] OA (10 μCi) and 1 μL of 100 mM OA thoroughly in a microcentrifuge tube.

55) Dry the mixture under a flow of N_2 gas at room temperature.

56) Add around 200 μL LD fractions (approximately 100 μg LD proteins) to the dried mixture in the tube.

▲ **CRITICAL STEP** Studying lipid synthesis on LDs requires a subpopulation of LDs with minimal contamination from other cellular organelles. The specific methodology for isolating this subpopulation can be found in our previous work[36].

? TROUBLESHOOTING

57) Incubate the sample at 4°C for 6 h to allow for the recruitment of OA by LDs from the surface of the tube..

58) Aliquot the above OA-associated LDs into five tubes.

59) Add 2 mM ATP and 2 mM CoA to the tube.

60) Incubate the tubes at 37°C for 0, 10, 30, 60, and 120 min respectively.

61) After the incubation, terminate the reaction immediately on ice and then re-isolate the LDs by centrifugation at 20,000*g* for 3 min.

62) Wash the isolated LDs with Buffer B three times.

63) Extract the total lipids from the LDs using 1 mL acetone/chloroform (2:1, v/v).

64) Centrifuge at 20,000*g* for 10 min to precipitate proteins and collect the organic supernatant.

65) Dry the supernatant under N_2 and dissolve the lipids in chloroform.

66) Analyze the extracted lipids by TLC using the following developing solvents, chloroform/acetone/methanol/acetic acid/water (80:40:30:20:10, v/v/v/v/v) for phospholipids and hexane/diethyl ether/acetic acid (80:20:1, v/v/v) for neutral lipids.

▲ **CRITICAL STEP** Additionally, less than 10 μL of lipids were loaded onto the plate at a time to minimize adsorption onto the tube wall.

67) After TLC separation, place the plates in an iodine vapor chamber for staining.

68) Once the TLC plates are dry at room temperature, scrape and collect the bands corresponding to the lipid standards that are separated on the same plate.

▲ **CRITICAL STEP** To avoid cross-contamination between different lipids, the blade should be cleaned with ethanol before scraping each band from the TLC plate.

69) Measure the radioactivity of the collected bands using a PerkinElmer scintillation counter.

LDs isolated from CHO K2 cells with minimal contamination from other organelles are found to actively incorporate [^{3}H] OA, demonstrating their intrinsic lipid synthesis capacity. This incorporation displays biphasic kinetics, suggesting an initial rapid phase utilizing pre-existing lipid intermediates followed by a slower phase limited by new synthesis. The fact that only ATP and CoA are required further supports LDs as sites for lipid synthesis (Supplemental Fig. 6). These findings highlight the role of LD as an active organelle, equipped with the necessary enzymes and substrates to contribute directly to cellular lipid metabolism.

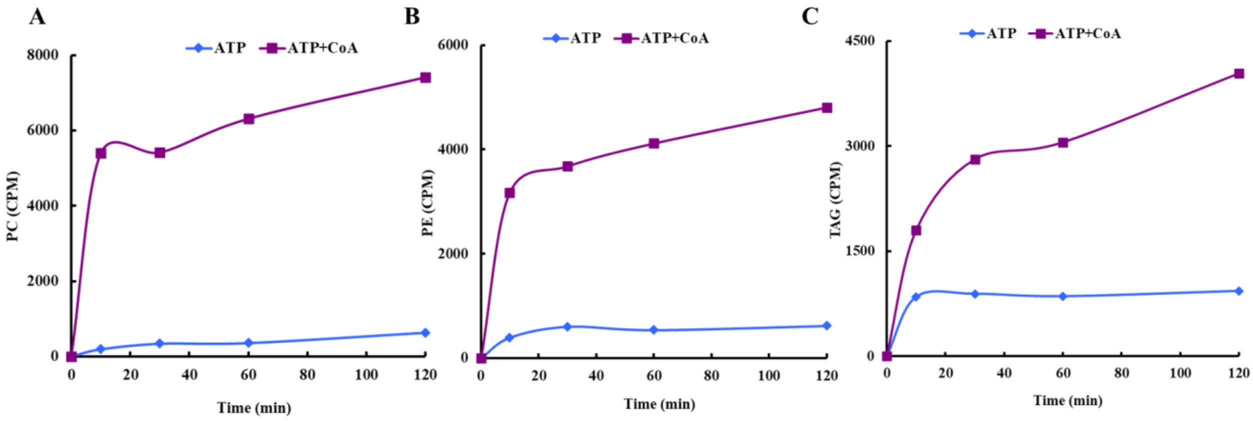

Supplemental Figure 6 The prepared OA solution is utilized for *in vitro* investigations into lipid synthesis mediated by ER-reduced lipid droplets.

The detailed procedure is described in the main text. Briefly, the LD subpopulation nearly depleted of ER, [^{3}H] OA and the prepared OA solution were used to investigate PC, PE, and TAG synthesis mediated by ER-depleted LDs *in vitro* in the presence of ATP and CoA (**A**, **B,** and **C**, respectively). The reactions exhibited saturation kinetics, which indicates the assay is effective. PC, phosphatidylcholine; PE, phosphatidylethanolamine; TAG, triacylglycerol. The figure is modified with permission from reference[36].

? TROUBLESHOOTING

Troubleshooting tips can be found in Supplemental Table 2.

Supplemental Table 2 Troubleshooting table.

Step	Problem	Possible reasons	Solution
4,5	Agglomerates form during sonication or white particles observed during loading into the working system	Sonication leads to an uneven or insufficient distribution of its effects	Use an ultrasonic homogenizer equipped with a thin probe (Fig. 1A)<br>Prolong sonication time<br>Move the tube up and down during sonication
12	The introduction of the FA solution results in the medium appearing cloudy	The medium has not reached a temperature of 60°C<br>FA is not sonicated thoroughly	Extend the duration for warming the medium<br>Repeat the sonication process for the FA, as described in Step 4
19	Phosphorylation of a protein can not be detected	The protein is highly dynamic or has low stability	Refer to our established protocol for preparing protein samples from both cells and tissues[40]
45	LDs with significant contaminations from other organelles are obtained	LDs may be broken during isolation<br>Subpopulation of LDs may be necessary	Refer to our established protocol and methodology for the LD purification and subpopulation[36, 41]

TIMING

Steps 1-3, before starting: ~ 4 min

Steps 4-5, ultrasonication: ~ 5 min

Step 6, assessment of the quality: variable

Step 7, storage: variable

Steps 8-10, usage: variable

Steps 11-69, downstream applications: variable

References

1. Siddiqui RA, Jenski LJ, Neff K, Harvey K, Kovacs RJ, Stillwell W: **Docosahexaenoic acid induces apoptosis in Jurkat cells by a protein phosphatase-mediated process.** *Biochim Biophys Acta* 2001, **1499:**265-275.
2. Cook HW, Clarke JT, Spence MW: **Involvement of triacylglycerol in the metabolism of fatty acids by cultured neuroblastoma and glioma cells.** *Journal of lipid research* 1982, **23:**1292-1300.
3. Huang S, Rutkowsky JM, Snodgrass RG, Ono-Moore KD, Schneider DA, Newman JW, Adams SH, Hwang DH: **Saturated fatty acids activate TLR-mediated proinflammatory signaling pathways.** *J Lipid Res* 2012, **53:**2002-2013.
4. Spector AA: **Structure and lipid binding properties of serum albumin.** In *Methods in Enzymology. Volume* 128: Academic Press; 1986: 320-339
5. Demant EJF, Richieri GV, Kleinfeld AM: **Stopped-flow kinetic analysis of long-chain fatty acid dissociation from bovine serum albumin.** *The Biochemical journal* 2002, **363:**809-815.
6. Listenberger LL, Ory DS, Schaffer JE: **Palmitate-induced apoptosis can occur through a ceramide-independent pathway.** *J Biol Chem* 2001, **276:**14890-14895.
7. Pardo V, Gonzalez-Rodriguez A, Guijas C, Balsinde J, Valverde AM: **Opposite cross-talk by oleate and palmitate on insulin signaling in hepatocytes through macrophage activation.** *J Biol Chem* 2015, **290:**11663-11677.
8. Schittmayer M, Birner-Gruenberger R: **Functional proteomics in lipid research: lipases, lipid droplets and lipoproteins.** *J Proteomics* 2009, **72:**1006-1018.
9. El-Assaad W, Buteau J, Peyot ML, Nolan C, Roduit R, Hardy S, Joly E, Dbaibo G, Rosenberg L, Prentki M: **Saturated fatty acids synergize with elevated glucose to cause pancreatic beta-cell death.** *Endocrinology* 2003, **144:**4154-4163.
10. Roche E, Buteau J, Aniento I, Reig JA, Soria B, Prentki M: **Palmitate and oleate induce the immediate-early response genes c-fos and nur-77 in the pancreatic beta-cell line INS-1.** *Diabetes* 1999, **48:**2007-2014.
11. Tracy LM, Bergqvist F, Ivanova EV, Jacobsen KT, Iverfeldt K: **Exposure to the saturated free fatty acid palmitate alters BV-2 microglia inflammatory response.** *J Mol Neurosci* 2013, **51:**805-812.
12. Wang Z, Liu D, Wang F, Liu S, Zhao S, Ling EA, Hao A: **Saturated fatty acids activate microglia via Toll-like receptor 4/NF-kappaB signalling.** *Br J Nutr* 2012, **107:**229-241.
13. Haversen L, Danielsson KN, Fogelstrand L, Wiklund O: **Induction of proinflammatory cytokines by long-chain saturated fatty acids in human macrophages.** *Atherosclerosis* 2009, **202:**382-393.
14. Lee JY, Sohn KH, Rhee SH, Hwang D: **Saturated fatty acids, but not unsaturated fatty acids, induce the expression of cyclooxygenase-2 mediated through Toll-like receptor 4.** *J Biol Chem* 2001, **276:**16683-16689.
15. Zhou YP, Grill VE: **Long-term exposure of rat pancreatic islets to fatty acids inhibits glucose-induced insulin secretion and biosynthesis through a glucose fatty acid cycle.** *J Clin Invest* 1994, **93:**870-876.
16. Chavez JA, Knotts TA, Wang LP, Li G, Dobrowsky RT, Florant GL, Summers SA: **A role for ceramide, but not diacylglycerol, in the antagonism of insulin signal transduction by saturated fatty acids.** *J Biol Chem* 2003, **278:**10297-10303.

17. Sofyana NT, Zheng J, Manabe Y, Yamamoto Y, Kishino S, Ogawa J, Sugawara T: **Gut microbial fatty acid metabolites (KetoA and KetoC) affect the progression of nonalcoholic steatohepatitis and reverse cholesterol transport metabolism in mouse model.** *Lipids* 2020.
18. Kim JY, Lee HJ, Lee SJ, Jung YH, Yoo DY, Hwang IK, Seong JK, Ryu JM, Han HJ: **Palmitic Acid-BSA enhances Amyloid-beta production through GPR40-mediated dual pathways in neuronal cells: Involvement of the Akt/mTOR/HIF-1alpha and Akt/NF-kappaB pathways.** *Sci Rep* 2017, **7:**4335.
19. Martin DD, Vilas GL, Prescher JA, Rajaiah G, Falck JR, Bertozzi CR, Berthiaume LG: **Rapid detection, discovery, and identification of post-translationally myristoylated proteins during apoptosis using a bio-orthogonal azidomyristate analog.** *Faseb j* 2008, **22:**797-806.
20. Yap MC, Kostiuk MA, Martin DD, Perinpanayagam MA, Hak PG, Siddam A, Majjigapu JR, Rajaiah G, Keller BO, Prescher JA, et al: **Rapid and selective detection of fatty acylated proteins using omega-alkynyl-fatty acids and click chemistry.** *J Lipid Res* 2010, **51:**1566-1580.
21. Erion KA, Berdan CA, Burritt NE, Corkey BE, Deeney JT: **Chronic Exposure to Excess Nutrients Left-shifts the Concentration Dependence of Glucose-stimulated Insulin Secretion in Pancreatic beta-Cells.** *J Biol Chem* 2015, **290:**16191-16201.
22. Song YF, Hogstrand C, Ling SC, Chen GH, Luo Z: **Creb-Pgc1alpha pathway modulates the interaction between lipid droplets and mitochondria and influences high fat diet-induced changes of lipid metabolism in the liver and isolated hepatocytes of yellow catfish.** *J Nutr Biochem* 2020, **80:**108364.
23. Las G, Serada SB, Wikstrom JD, Twig G, Shirihai OS: **Fatty acids suppress autophagic turnover in beta-cells.** *J Biol Chem* 2011, **286:**42534-42544.
24. Malhi H, Bronk SF, Werneburg NW, Gores GJ: **Free fatty acids induce JNK-dependent hepatocyte lipoapoptosis.** *J Biol Chem* 2006, **281:**12093-12101.
25. Akazawa Y, Cazanave S, Mott JL, Elmi N, Bronk SF, Kohno S, Charlton MR, Gores GJ: **Palmitoleate attenuates palmitate-induced Bim and PUMA up-regulation and hepatocyte lipoapoptosis.** *J Hepatol* 2010, **52:**586-593.
26. Wen H, Gris D, Lei Y, Jha S, Zhang L, Huang MT, Brickey WJ, Ting JP: **Fatty acid-induced NLRP3-ASC inflammasome activation interferes with insulin signaling.** *Nat Immunol* 2011, **12:**408-415.
27. Spector AA, John KM: **Effects of free fatty acid on the fluorescence of bovine serum albumin.** *Arch Biochem Biophys* 1968, **127:**65-71.
28. Liu P, Ying Y, Zhao Y, Mundy DI, Zhu M, Anderson RG: **Chinese hamster ovary K2 cell lipid droplets appear to be metabolic organelles involved in membrane traffic.** *J Biol Chem* 2004, **279:**3787-3792.
29. Peng G, Li L, Liu Y, Pu J, Zhang S, Yu J, Zhao J, Liu P: **Oleate blocks palmitate-induced abnormal lipid distribution, endoplasmic reticulum expansion and stress, and insulin resistance in skeletal muscle.** *Endocrinology* 2011, **152:**2206-2218.
30. Chen X, Xu S, Wei S, Deng Y, Li Y, Yang F, Liu P: **Comparative Proteomic Study of Fatty Acid-treated Myoblasts Reveals Role of Cox-2 in Palmitate-induced Insulin Resistance.** *Sci Rep* 2016, **6:**21454.
31. Cui L, Mirza AH, Zhang S, Liang B, Liu P: **Lipid droplets and mitochondria are anchored**

during brown adipocyte differentiation. *Protein Cell* 2019, **10:**921-926.

32. Liu Y, Xu S, Zhang C, Zhu X, Hammad MA, Zhang X, Christian M, Zhang H, Liu P: **Hydroxysteroid dehydrogenase family proteins on lipid droplets through bacteria, C. elegans, and mammals.** *Biochim Biophys Acta Mol Cell Biol Lipids* 2018, **1863:**881-894.
33. Pu J, Peng G, Li L, Na H, Liu Y, Liu P: **Palmitic acid acutely stimulates glucose uptake via activation of Akt and ERK1/2 in skeletal muscle cells.** *J Lipid Res* 2011, **52:**1319-1327.
34. Huang T, Bamigbade AT, Xu S, Deng Y, Xie K, Ogunsade OO, Mirza AH, Wang J, Liu P, Zhang S: **Identification of noncoding RNA-encoded proteins on lipid droplets.** *Science Bulletin* 2021, **66:**314-318.
35. Yuan Q, Zheng K, Huang T, Zhi Z, Xu Y, Cui L, Liu P, Zhang S: **One amino acid drives the lipid droplet targeting sequence of a new noncoding RNA-encoded protein to mitochondrion.** *Proteomics* 2023, **23:**e2200301.
36. Zhang S, Wang Y, Cui L, Deng Y, Xu S, Yu J, Cichello S, Serrero G, Ying Y, Liu P: **Morphologically and Functionally Distinct Lipid Droplet Subpopulations.** *Sci Rep* 2016, **6:**29539.
37. Zhang H, Wang Y, Li J, Yu J, Pu J, Li L, Zhang H, Zhang S, Peng G, Yang F, Liu P: **Proteome of skeletal muscle lipid droplet reveals association with mitochondria and apolipoprotein a-I.** *J Proteome Res* 2011, **10:**4757-4768.
38. Deng Y, Zhou C, Mirza AH, Bamigbade AT, Zhang S, Xu S, Liu P: **Rab18 binds PLIN2 and ACSL3 to mediate lipid droplet dynamics.** *Biochim Biophys Acta Mol Cell Biol Lipids* 2021, **1866:**158923.
39. Fu X, Zhang S, Liu P: **Co-immunoprecipitation for identifying protein-protein interaction on lipid droplets.** *Biophys Rep* 2024, **10:**102-110.
40. Cheng L, Xu Y, Zhu K, Liang B, Zhang S, Liu P: **Protein sample preparation for tissue distribution study.** *Proteomics Clin Appl* 2023, **17:**e2200088.
41. Ding Y, Zhang S, Yang L, Na H, Zhang P, Zhang H, Wang Y, Chen Y, Yu J, Huo C, et al: **Isolating lipid droplets from multiple species.** *Nat Protoc* 2013, **8:**43-51.
